# The Modification of Dietary Protein with Ammonium Hydroxide Enhancement Improves Longevity and Metabolic Outcomes in a Sex-Dependent Manner

**DOI:** 10.3390/nu16162787

**Published:** 2024-08-21

**Authors:** Benjamin Barr, Lauren Gollahon

**Affiliations:** 1 Department of Biological Sciences, Texas Tech University, 2500 Broadway, Lubbock, TX 79409, USA; benjamin.barr@ttu.edu; 2 Obesity Research Institute, Texas Tech University, 2500 Broadway, Lubbock, TX 79409, USA

**Keywords:** casein, beef, longevity, high-fat diet, ammonium hydroxide, dietary proteins, cross-sectional studies, obesity

## Abstract

(1) Background: Dietary protein is a key component of all dietary patterns. It has been demonstrated that there are subtle differences in health implications associated with the source of dietary protein consumed. This study examined dietary protein sources (DPSs) in a long-term study of diet-induced obesity ± ammonium hydroxide enhancement (AHE) and its role in improving long-term health outcomes. (2) Methods: Over 18 months, 272 C3H/HeJ mice (136 male and 136 female) were monitored on high-fat diets with varying DPSs ± AHE. Mice were monitored for weekly change in total mass, as well as 6-month assessments of lean and fat mass. At each assessment, a cohort (~8 mice per diet per sex) was censored for a cross-sectional examination of organ function. (3) Results: Longevity was improved in females fed AHE diets, regardless of DPSs. Females’ measures of fat and lean mass were markedly elevated with casein protein diets compared to beef protein diets regardless of AHE. Females fed a beef protein diet + AHE demonstrated reduced fat mass and increased lean mass with aging. In males, AHE beef protein diet-fed mice showed marked improvement to longevity and increased lean mass at 6 months. (4) Conclusions: This study demonstrates that dietary protein modification by AHE attenuates the negative impacts of HF diets in both males and females in a sex-dependent manner. Furthermore, the results from this study emphasize the importance of identifying the differences in the utilization of dietary proteins in both a sex- and age-related manner and demonstrate the potential of DPS modification by AHE as a dietary intervention.

## 1. Introduction

The global obesity epidemic is increasingly being attributed to dietary habits associated with the Western Dietary (WD) pattern [1,2]. Obesity is a major contributor to the development of further chronic conditions, such as metabolic syndrome (MetS) and cancer, along with more tissue-specific diseases such as heart disease and metabolic-associated fatty liver disease (MAFLD) [3,4]. Currently, most preclinical studies of obesity examine the disease in an acute fashion, rapidly inducing obesity in animal models over ~12 weeks, rarely following the subjects past the initial ~12-week period [5,6,7,8]. While yielding important insights, this approach also generates a gap in understanding, since human obesity develops as the result of years of progressive weight gain [9]. As a chronic condition, short-term mouse models may miss subtle but critical changes in the etiology of the obese condition, especially in the context of diet-induced obesity (DIO).

The WD is one of the most examined methods of DIO in preclinical studies [10]. Typically consisting of some combination of high-fat (HF) and high-sugar diet for preclinical models, this reflects the WD consumed by humans [10]. Furthermore, these diets are presented in preclinical models ad libitum, an availability similar to the WD consumption pattern and sedentary lifestyle [1,2,10]. The WD is characterized by its composition of large amounts of processed meats, fats, and sodium with lower amounts of fiber and potassium [11]. The treatment of conditions like obesity and MetS are regularly focused on restricting total caloric intake, usually in terms of fats and sugars [12]. However, substantial evidence exists to support the consumption of increased levels of dietary protein to reduce body weight and fat mass [13], and data suggest that increases in dietary protein reduce body weight by modulating systemic energy metabolism and appetitive signaling [13]. Significantly fewer studies explore the contributions of proteins, specifically animal proteins, to life expectancy, the gut microbiome, and the development of dense tissue, metabolic-associated diseases [11]. A high consumption of dietary protein is often associated with chronic, systemic acidosis as a result of its net endogenous acid production (NEAP) or the amount of acids and bases produced in the body [11]. NEAP estimates acid load specific to diet composition [14,15,16]. Meat, animal products, and grains are generally considered to be net acid-producing [11]. In contrast, vegetables and most fruits are associated with net base production. Since animal-derived proteins include sulfur-containing amino acids (these are sometime missing in plant-based foods), they are a potential source of exogenous acids. Fruits and vegetables are considered precursor sources for base production [11]. Studies demonstrate that these trends in WD components can impact pH homeostasis at the cellular level [17], contributing to the downstream effect of diagnosed metabolic diseases.

While meat and animal products containing some fat are not unhealthy, when combined with a diet high in other nutrient-poor foods, these factors can lead to disruptions to cellular pH homeostasis [17]. Very few long-term studies have been performed that include modifications to the pH of specific dietary components or the diet as a whole. Most information regarding differences in nutritional protein consumption is focused on muscle and exercise nutrition, emphasizing which protein components (amino acids or peptides) are beneficial to the muscle building process and most important for decreasing muscle breakdown [18]. Examples of these are branched chain amino acids such as leucine, isoleucine, and valine [18]. However, information regarding the role of different dietary protein sources (DPSs) is not well established [19].

Protein is generally considered the same, only differing between animal-based and plant-based. Recently, a study was performed examining the effects of dietary amino acid sources by using a free amino acid mixture mimicking casein (milk protein) to compare against a casein-based diet [20]. The study reported that metabolically associated outcomes, such as non-alcoholic fatty liver disease (NAFLD, now referred to as MAFLD), were decreased in the free amino acid mixture, indicating that DPSs and their formulation are crucial to overall health [20]. Animal protein supports lean muscle mass gain and maintenance more than plant-based protein, which lacks anabolic potential [21]. Meat protein is recommended for elderly individuals trying to maintain weight and muscle mass [22] and iron and zinc as well as heme iron and heme zinc are significantly more bioavailable in red meat than any other source [23].

With the current research initiative of “Food is Medicine” introduced by the Department of Health and Human Services [24], this study conducted a long-term investigation into the influence of an FDA-approved [25] method for dietary protein modification. We hypothesized that modifying the pH of the protein component prior to addition to a complete diet would mitigate the acidic effects of an HFD towards inducing MAFLD and liver cancer. This hypothesis was based on the rationale that ammonium hydroxide enhancement (AHE), an FDA-approved step in beef processing [25], could be used to raise the pH of the protein; then, this protein could be reincorporated into an HFD to improve health outcomes. Digestion in the gut favors ammonium hydroxide formation as gut pH is lower than the pKa of ammonia (~9.2), and the equilibrium equation shifts right [11]. While hydroxyl is oxidizing lipids, excess ammonia inhibits anaerobic metabolism in pathogenic gut microbial species [26,27]. Physiological evidence supporting an alkaline shift in the diet includes decreased cortisol secretion by reducing or neutralizing dietary load [28], alkalinizing the tumor microenvironment enhancing tumor response to chemotherapy [29], and higher levels of plasma bicarbonate associated with lower risk for type 2 diabetes [30]. The digestion of more alkaline foods into their functional molecules may involve more physiological and cellular factors that counterbalance metabolic acidosis-associated disease. For example, adding more fruits and vegetables into a WD reduces cardiovascular risk associated with chronic kidney disease more effectively than sodium bicarbonate treatment alone [31]. Furthermore, the digestion and absorption of certain foods can maintain or help regain metabolic balance at the cellular and physiological levels by increasing the bioavailability of ammonia-associated metabolites that can involve many pathways.

In a preclinical study using C57Bl/6 mice, it was shown that AHE provided metabolic benefits in males, but effects in females were unclear as they did not develop obesity [7]. Additional research is required to explore the nuances of sexual dimorphisms surrounding dietary protein, especially in diseases that are already sexually dimorphic, such as MAFLD. Component-based dietary interventions have yet to be explored as methods to combat the obesity epidemic, but such interventions would minimally impact established dietary patterns while improving metabolic measures. In this study, we explored the use of AHE as a method to modify DPSs to improving metabolically associated health outcomes in a chronic setting. To that end, we hypothesized that AHE in beef protein in an HFD would improve longevity, reduce obesity, and improve liver health in C3H/HeJ regardless of sex. Assessments were made to reflect the chronic nature of DIO and its association with aging. To that end, liver health (associated heavily with lipid formation), weight gain, and obesity were measured over time, and variations between dietary groups, sexes, and ages were analyzed. Overall, this dietary intervention approach aims to establish a method to improve metabolic health outcomes, without drastically altering established dietary patterns.

## 2. Materials and Methods

### 2.1. Animal Study and Diets

The selection of the C3H/HeJ mouse strain was based on disease susceptibility, lifespan, and less aggressive social behavior [32]. Mouse handling followed regulatory compliance at all times under the approved Texas Tech University IACUC protocol number 19021-02. Similar to our previous study [33], 272 C3H/HeJ mice were obtained from Jackson Laboratories (Bar Harbor, ME, USA) at 4 weeks old. After one-week acclimatization, mice were randomly separated by cage into the following diet groups: high-fat casein (HFC) (460 kcal/100 g, 46% energy as fat), high-fat casein diet with AHE (HFCN) (460 kcal/100 g, 46% energy as fat), high-fat beef protein (HFB) (470 kcal/100 g, 46% energy as fat), and high-fat beef protein with AHE (HFBN) (470 kcal/100 g, 46% energy as fat). All diets were formulated by Research Diets, Inc. (New Brunswick, NJ, USA). Dietary components are listed in detail in Table S1. Females and males were housed 4 per cage, in separate temperature-, humidity-, and 12 h light and 12 h dark cycle-controlled rooms. Animals had access to water and their assigned dietary group in pellet form, ad libitum. At collection time points and at the termination of this study, mice were euthanized after a 1 h fast, using CO_2_ followed by cervical separation. The baseline group consisted of 8 male and 8 female mice, which were euthanized after the one-week adjustment period. The long-term study groups (consisting of 32 mice each) were maintained on high-fat (46% kcal) diets ± AHE for up to 18 months. Weekly measurements of individual weights, food consumption per cage, and photographic records of individuals were performed. Monthly, measurements to assess changes in fat and lean mass were performed using EchoMRI (EchoMRI, Houston, TX, USA). Terminal cross-sectional tissue collections (censoring) were performed every 6 months on ~8 mice (~1/4 of each group), where mice were euthanized using CO_2_.

### 2.2. Histology

Immediately following euthanasia, tissues from the brain, heart, lung, liver, kidneys, visceral fat, stomach, small intestines, reproductive organs, and skeletal muscle were collected. A portion of the tissue of interest and any observed tumors were fixed in 10% formalin and processed for embedding. The tissues were embedded in paraffin using a Leica EG1160 tissue embedding station (Leica, Nussloch, Germany), and 5 µm sections were cut. Upon obtaining three clean serial sections, one section from each tissue was stained with Mayer’s hematoxylin and alcoholic eosin Y (H&E) following standard practice [34]. After the standard dewaxing and dehydration of sections, specific staining procedures included initial staining with hematoxylin (15 min), rinsing in both bluing solution (1 min in 0.01% NaCO_3_) and running tap water (15 min), then a wash in 95% ethanol (1 min) followed by eosin staining (1 min), and a final 80% ethanol wash (1 min) before finishing with standard steps from dehydration to cover-slipping.

### 2.3. Statistical Analysis

Full code and raw data can be found at https://github.com/BenjaminBarr/HF-and-AHE-Diet/releases/tag/v1.1 (accessed on 20 August 2024). A modified Kaplan–Meier graph was generated to show differences in longevity between collection points in R (v4.3.1) [35], using the survival package (v1.1.0) and ggsurvfit (v3.6-4) [36,37]. Significance was assessed using the log-rank test (Mantel–Haenszel) [37]. Post hoc analysis was not performed. An ANOVA was performed with R to assess mass and body composition, a fixed-effects linear model with sex, and diet at each time point, via lme4 (v1.1-35.3) [38]. To detect the differences between dietary protein content, AHE, sex, and their combination, emmeans (v1.10.2) was used with Šídák pair-wise comparisons (post hoc) and a Family-wise error rate at 0.05 [39]. Šídák was chosen over Tukey for post hoc analysis because it is similar to Bonferroni but assumes each comparison is independent. It is the least conservative method and assumes that each comparison is independent of the others. Šídák can be used to determine statistical significance, calculate adjusted *p*-values, and compute confidence intervals when comparing a group of independent comparisons (i.e., two or more groups across a range of metrics). All results are presented as the mean ± SEM (standard error of mean), with statistical significance considered at *p* ≤ 0.05. Interactions are fully displayed in Supplementary Table S2.

### 2.4. Lipid Droplet Size Assessment Trainable Weka Workflow

Lipid droplet (LD) perimeter was assessed with the aid of WEKA Segmentation in FIJI. The full workflow can be found in the Supplementary Materials (Figure S1). This semi-automated method allows for the rapid identification of lipid droplets with flexibility for the researcher to modify criteria for selection on a “per slide” basis. It is important that the user is experienced in tissue morphology as physical characteristics such as hepatocyte ballooning and occult tumors can confound the results.

Steatosis was assessed in H&E sectioned livers at 10× magnification. LDs were selected into class 1, based on size, shape, color, and circularity. Hepatocytes, other typical liver structures (e.g., central veins, and portal triads), and the slide background were selected into class 2. LDs were measured by area, circularity (0.5–1; 1 = perfect circle), and perimeter. To filter out noise, any region of interest (ROI) with an area <1 µm were excluded from the data. LD measurements were pooled by sex, group, and time before being averaged and graphed using ggplot in R. Notes were added indicating hepatocyte ballooning (Yes/No regardless of intensity) and the presence/absence of LDs for each slide.

## 3. Results

### 3.1. Weekly Change in Total Mass

**Figure 1:**
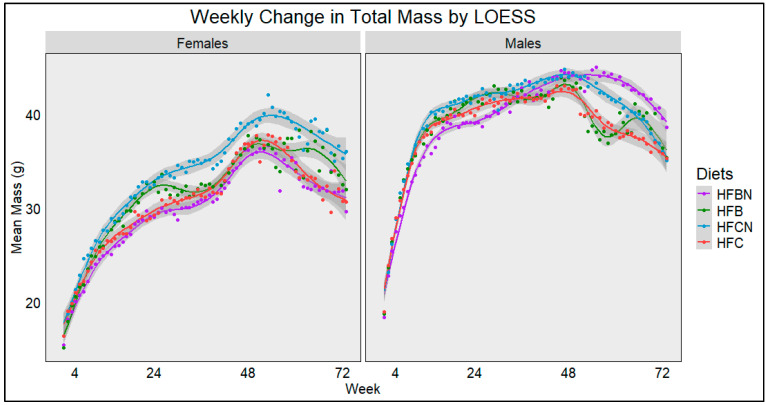
LOESS representations of weekly trends in mass over 72 weeks (18 months). Colors represent diet groups as follows: red = HFC, blue = HFCN, green = HFB, and purple = HFBN. Line shadows represent 0.95 CI for the estimation (*n* = 7–32, based on age/censorship).

The average weekly mass from each diet and sex was plotted using Locally Estimated Scatterplot Smoothing (LOESS) (Figure 1). Lines were generated using all the available data points close to that coordinate, and dots were added to indicate the actual mean total mass from each diet. The sample size ranged from 32 at time 0 to 7 at week 72. The gray bands indicate SE calculated by R for LOESS. Figure 1 demonstrates a rapid increase in total mass for females up until ~20 weeks. In contrast, males began to reduce their rate of total mass increase ~16 weeks. In both sexes, these trends continued regardless of protein ± AHE. After this point, the female mice began to noticeably diverge, with the HFCN group increasing in total mass most rapidly and maintaining the highest total mass throughout this study. The HFBN females maintained the lowest total mass until the last few weeks of this study. At ~48–52 weeks, mass for all female groups began to rapidly increase, before reaching highest total mass for the entire study. In males, HFBN males had the lowest total mass until ~30 weeks. Then, between 30 and 48 weeks, HFBN male mass increased to the highest total mass. This observation continued for the remainder of this study. Of note, the HFBN group maintained their mass until ~60 weeks, while all other groups began to decline in mass after ~48 weeks. The most dramatic declines occurred in the unenhanced groups (HFC and HFB). Compared with the other dietary groups, the HFB group also demonstrated a fluctuation in weight in the second half of the experiment. Following the initial decrease in total mass after ~48 weeks, mass increased then decreased rapidly before increasing then decreasing again just prior to experiment termination (Figure 1, right panel).

### 3.2. Kaplan–Meier Analysis of C3H/HeJ Mice Maintained on High-Fat Beef or Casein Protein ± AHE

**Figure 2:**
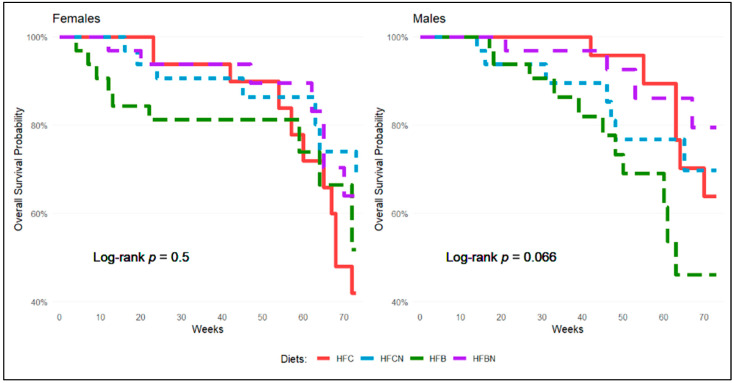
Kaplan–Meier survival assessment over study duration for male and female mice maintained on HF diets ± AHE. Colors represent diet groups as follows: red = HFC, blue = HFCN, green = HFB, and purple = HFBN (*n* ≤ 32). Log-rank test for significance was conducted separately for each sex. Results showed Chi-square = 2.5 on 3 degrees of freedom and assessed for *p*-value > 0.1, α = 0.1 for females (left panel). Results showed Chi-square = 7.2 on 3 degrees of freedom and assessed for *p*-value > 0.1, α = 0.1 for males (right panel).

Kaplan–Meier analysis was used to assess differences in survivability between dietary groups within the same sex over the course of this study. Figure 2 shows survival differences based on diet and separated by sex. In females (Figure 2, left panel), all diets except the HFB diet maintained similar survivability until ~54 weeks. At this point, a rapid increase in deaths (events) was observed in the HFC diet-fed females. Females maintained on the AHE diets (HFCN and HFBN) demonstrated similar survivability over the entire study. The rapid declines demonstrated initially by the HFB diet-fed group (~20 weeks) and later by the HFC diet-fed group (~70 weeks) are time frames where survivability drastically decreased.

For males (Figure 2, right panel), the HFB diet-fed group demonstrated a consistently lower survival rate than all other groups. HFC and HFBN diet-fed groups exhibited similar survivability until ~42 weeks. After this point, deaths in both groups increased, with the HFC group showing an even greater decline in survivability at ~60 weeks. Overall, the HFBN and HFCN diet-fed mice demonstrated similar gradual decreases in survivability over the course of this study, with HFBN diet-fed mice demonstrating the highest survivability by the end of this study. Differences between survivability in the HFBN and HFB diet-fed males are significant at α ≤ 0.1, as demonstrated by the log-rank test and *p* = 0.065. Post hoc analysis was not perfor

### 3.3. Analysis of Female Mass Composition Compared with T0 Baseline

When compared to time 0 baseline individuals, the females maintained on casein, regardless of AHE, had increases in fat mass of more than 1000% (1063% and 1280% for HFC and HFCN diet-fed females, respectively). Interestingly, females in the HFB and HFBN diet-fed groups had maximal increases of 705% and 814%, respectively. This change occurred at 12 months for all diet-fed groups except the HFB diet-fed group, in which the change occurred at 18 months. The maximal lean mass for the HFC diet-fed group was 53%, HFCN diet-fed group was 55%, HFB diet-fed group was 52%, and HFBN diet-fed group was 29%. These increases were present at 12 months for females maintained on casein (HFC and HFCN), at 18 months for the HFB diet-fed group, and at 6 months for the HFBN diet-fed group. It is important to note that discrepancies in the sums of average fat and lean mass are generally attributable to water weight, which is not recorded here. However, in the cases of the HFC diet-fed females at 6 months and the HFB diet-fed females at 12 months, many mice were unable to fit into the more constrictive MRI chamber. Thus, the next chamber size up was utilized, and it proved to be too large. As a result, mouse mass type measurements may be under-reported. Total mass in all groups increased from T0 between ~75 and 150% depending on the time point at which it was measured, and mass was always maximally elevated at 12 months. For females, the group with the highest total mass was the HFCN group. The results for the analysis of female mass composition are presented in Table 1.

### 3.4. Analysis of Male Mass Composition Compared with T0

When compared to time 0 baseline individuals, the males maintained on HF reference diets regardless of protein type had maximal increases in fat mass from T0, 667.36% and 552.08% (HFC and HFB, respectively), occurring at 6 months. In the AHE diet-fed groups (HFCN and HFBN), the maximal changes from T0 occurred at 12 months and were 753.47% and 640.97%, respectively. Table 2 summarizes the results for male mass composition. For lean mass, the casein diet-fed groups (HFC and HFCN) showed maximal changes at 12 months of 54.64% and 56.21%, respectively. The beef diets displayed a much more divergent trend, with the maximal lean mass in the HFBN diet-fed group occurring at 6 months with 62.28% and the HFB diet-fed group's maximal change in lean mass occurring at 18 months with 47.46%. Regardless of time point, HFBN diet-fed males demonstrated the highest lean mass change from T0 when compared with other groups. Similar to females, any disparities in the sum of average fat mass and average lean mass with regard to the total mass are likely the result of water weight, which is not shown here. Males maintained on AHE diets, HFCN and HFBN, demonstrated the highest total mass at 12 months. In contrast, the HFC and HFB groups’ total mass was most elevated at 6 months. The HFBN males demonstrated the highest total mass overall.

### 3.5. Age-Associated Change in Female Fat and Lean Mass Depots

**Figure 3:**
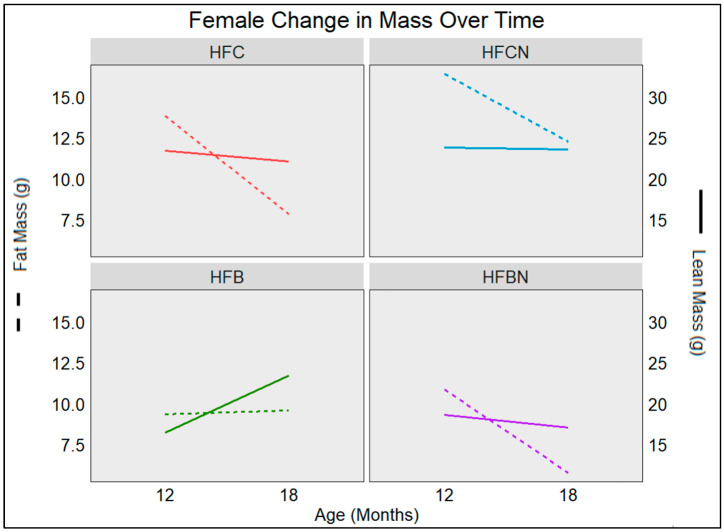
Visualization of changes in mass retention as the result of aging in C3H females. Measurements were taken at 12 and 18 months by Echo MRI. Solid lines represent change in lean mass (right y-axis), and dashed lines represent change in fat mass (left y-axis). Sample sizes (*n* > 7), and SE are reported in Table 1 for females.

Changes in females associated with aging (from 12 to 18 months) for each mass type (lean or fat) are illustrated in Figure 3. Females maintained on casein diets ± AHE tended to lose mass during this time frame. HFC diet-fed mice lost ~6 g of fat and ~1 g of lean mass, while HFCN diet-fed mice lost ~4 g of fat and only ~0.2 g of lean mass. While both groups had similar amounts of lean mass, the HFC diet-fed group lost more. Although the HFCN diet-fed group had more fat mass, this was not shed as rapidly as the HFC diet-fed group. The high-fat beef diets ± AHE both had lower fat and lean mass than their casein counterparts, regardless of age. Similarly, at 12 months, lean mass was lower in beef diets than in casein. This trend continued for the HFBN diet-fed females at 18 months, but in the HFB diet-fed group, lean mass dramatically increased during this time frame (12–18 months), becoming more reflective of the lean mass observed in the HFCN diet-fed mice. All groups, except the HFB group, decreased in fat (~5 g) and lean (~1 g) mass from 12 to 18 months. During this time, the HFB group increased in fat (~0.2 g) and lean (~7 g) mass.

### 3.6. Age-Associated Change in Male Mass Fat and Lean Mass Depots

**Figure 4:**
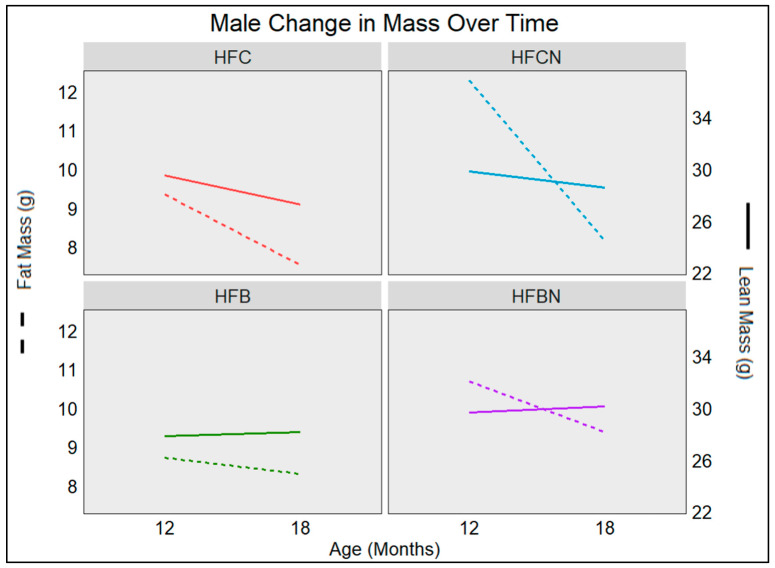
Visualization of changes in mass retention as the result of aging in C3H males. Measurements were taken at 12 and 18 months by Echo MRI. Solid lines represent change in lean mass (right *y*-axis), and dashed lines represent change in fat mass （left *y*-axis). Sample sizes (*n* > 4) and SE are reported in Table 2 for males.

Changes in male mass type, associated with aging, are illustrated in Figure 4. Males maintained on casein diets ± AHE lost mass during this time frame. HFC diet-fed mice lost ~1.8 g of fat and ~2.2 g of lean mass. HFCN diet-fed mice lost ~4 g of fat and only ~1.2 g of lean mass. While both groups showed similar amounts of lean mass, the HFCN diet-fed mice lost less of their lean mass while losing more of their fat mass than HFC diet-fed mice. Interestingly, both beef diets demonstrated slight decreases in fat mass, HFB ~0.4 g and HFBN ~0.7 g, while gaining lean mass ~0.3 g (HFB) and ~0.5 g (HFBN).

### 3.7. Assessment of Echo MRI Mass Composition in C3H/HeJ Females Using Two-Way ANOVA on Fixed-Effects Model

**Figure 5:**
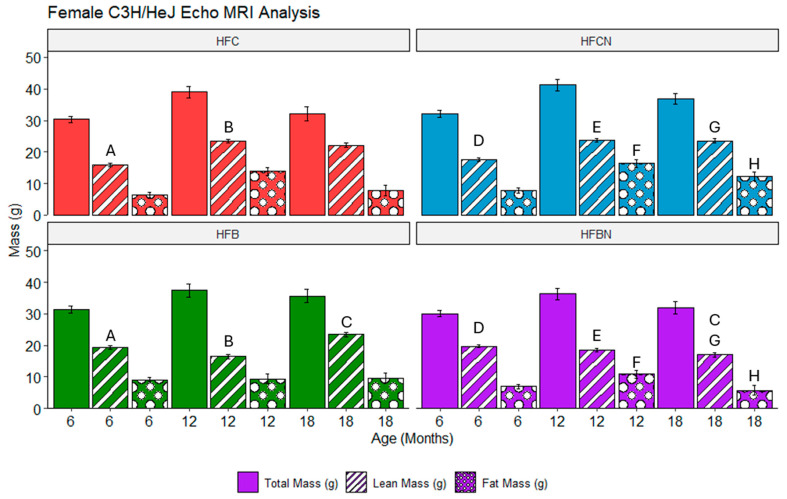
Analysis of mass composition depots in female mice for diets ± AHE, protein, and sex by three-way ANOVA. Differences are shown between each diet at specific time points for females. Significance is indicated by matching letters (A is significantly different from A) and only with *p*-values ≤ 0.05. Pair-wise comparisons are only displayed within sexes. Colors represent diet groups as follows: red = HFC, blue = HFCN, green = HFB, and purple = HFBN. Solid bars indicate total mass, striped bars indicate lean mass, and dotted bars indicate fat mass. Note: Significance was not assessed between time points.

Overall, the majority of differences identified in females are the result of DPSs (beef or casein). At 6 and 12 months, HFC and HFB diet-fed females demonstrated significantly different lean mass compositions when compared. At 6 months, the HFB diet-fed mice had higher lean mass than the HFC diet-fed group. However, the 12-month comparison showed that this result had flipped, with HFC diet-fed mice demonstrating greater lean mass than the HFB diet-fed mice. Interestingly, the HFCN and HFBN diet-fed groups demonstrated the same pattern with regards to lean mass; at 6 months, the mass of HFBN diet-fed mice was markedly elevated, and then at 12 months, HFCN diet-fed mice had markedly elevated lean mass. Furthermore, this difference is consistent throughout the remainder of this study (i.e., 18-months HFCN diet-fed mice had significantly elevated lean mass compared with HFBN diet-fed mice). At 12 and 18 months, the HFCN diet-fed females also demonstrated significantly elevated fat mass when compared with HFBN diet-fed groups. The only significant difference between AHE and HF reference diets (non-AHE) was identified at 18 months between the fat mass of the HFB and HFBN diet-fed groups. It is also important to note that there were no significant differences in total mass identified. These results are shown in Figure 5.

### 3.8. Assessment of Echo MRI Mass Composition in C3H/HeJ Males Using Three-Way ANOVA on Fixed-Effects Model

**Figure 6:**
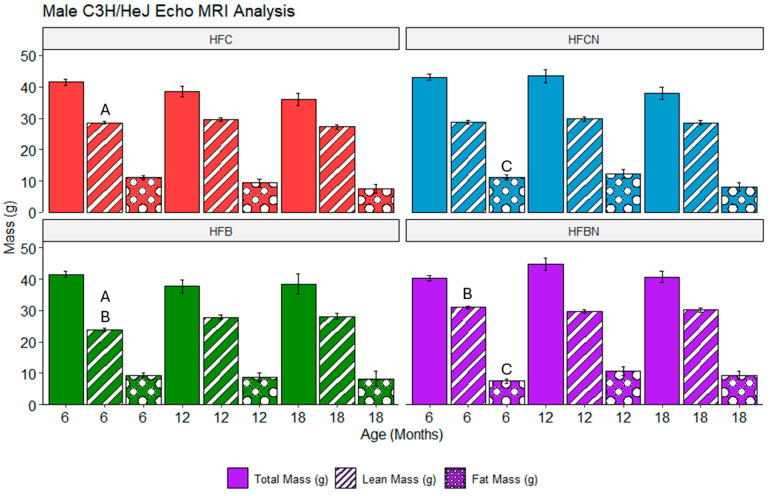
Analysis of mass composition in male mice for diets ± AHE, protein, and sex by three-way ANOVA was performed. Figure shows differences between each diet at specific time points for males. Significance is indicated by matching letters (A is significantly different from A) and only with *p*-values ≤ 0.05. Pair-wise comparisons are only displayed within sexes. Colors represent diet groups as follows: red = HFC, blue = HFCN, green = HFB, and purple = HFBN. Solid bars indicate total mass, striped bars indicate lean mass, and dotted bars indicate fat mass. Significance was not assessed between time points.

In males, fewer differences in mass composition were observed between the different diet groups. The results are shown in Figure 6. In the reference diets, the HFC diet-fed group 3 demonstrated markedly elevated lean mass at 6 months when compared with the HFB diet-fed group. This difference resolved by 12 months, and there were no further notable differences between these groups. In the AHE diets, the HFCN diet-fed group had markedly elevated fat mass when compared with the HFBN diet-fed group. Similar to lean mass in the reference groups, this disparity was resolved by 12 months. Between beef diets, lean mass was markedly elevated in the HFBN diet-fed group at 6 months when compared with the HFB diet-fed mice. Following the same pattern as the other differences, this disparity disappeared by 12 months.

### 3.9. Lipid Droplet Liver Infiltration (Steatosis) Assessed Using Weka Segmentation

**Figure 7:**
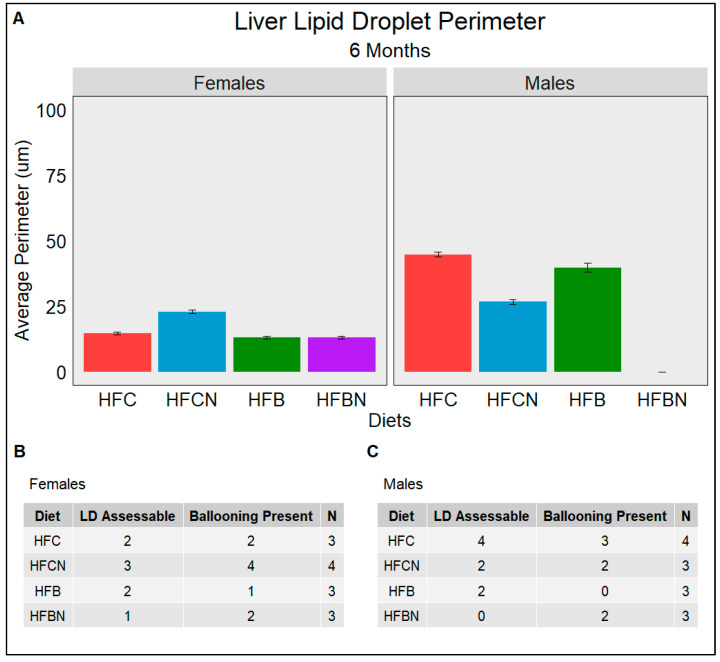
An assessment at 6 months of the average perimeter of LDs (Steatosis) identified in each dietary group separated by sex. (**A**) gives the average perimeter in µm of LDs assessed from H&E-stained liver slides. (**B**,**C**) The number of slides which had assessable LDs (LD Assessable) and the number of slides with hepatocyte ballooning (Ballooning Present). (**B**) females and (**C**) males. N is the total number of different slides viewed. All slides are from different mice; if LD was not indicated in a slide, it was either due to the absence of LD or excessive ballooning restricting FIJI’s ability to identify LD.

**Figure 8:**
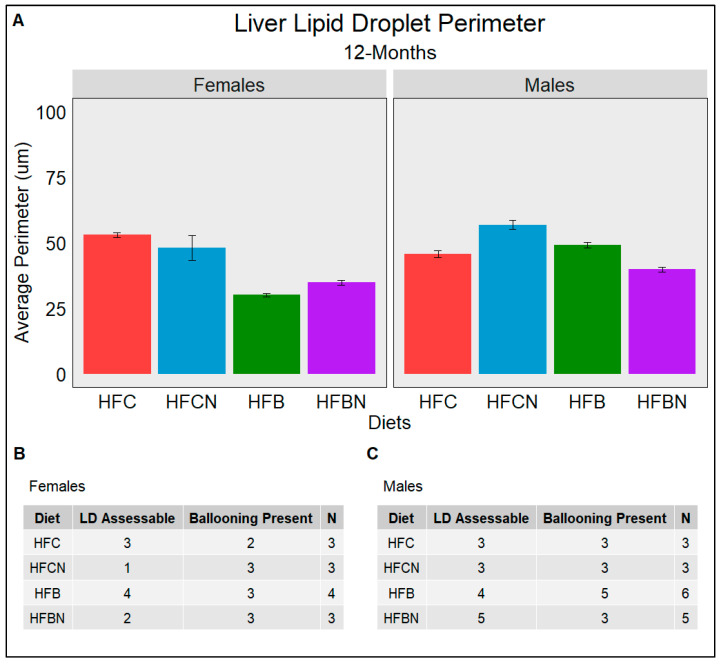
An assessment at 12 months of the average perimeter of LDs (Steatosis) identified in each dietary group separated by sex. (**A**) gives the average perimeter in µm of LDs assessed from H&E-stained liver slides. (**B**,**C**) The number of slides which had assessable LDs (LD Assessable) and the number of slides with hepatocyte ballooning (Ballooning Present). (**B**) females and (**C**) males. N is the total number of different slides viewed. All slides are from different mice; if LD was not indicated in a slide, it was either due to the absence of LD or excessive ballooning restricting FIJI’s ability to identify LD.

**Figure 9:**
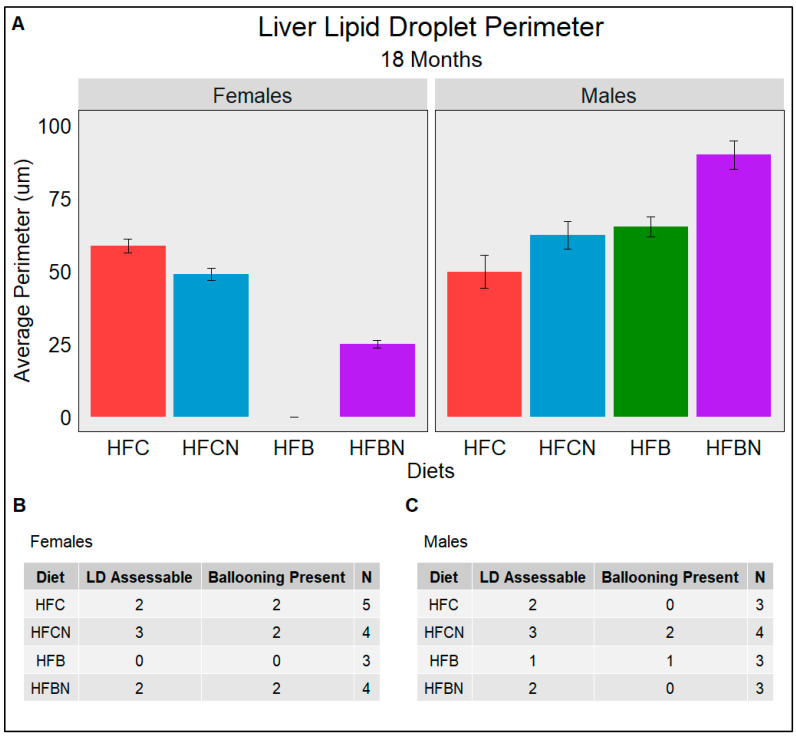
An assessment at 18 months of the average perimeter of LDs (Steatosis) identified in each dietary group separated by sex. (**A**) gives the average perimeter in µm of LDs assessed from H&E-stained liver slides. (**B**,**C**) The number of slides which had assessable LDs (LD Assessable) and the number of slides with hepatocyte ballooning (Ballooning Present). (**B**) females and (**C**) males. N is the total number of different slides viewed. All slides are from different mice; if LD was not indicated in a slide, it was either due to the absence of LD or excessive ballooning restricting FIJI’s ability to identify LD.

Steatosis is the major indicator of progression from a healthy liver into diseased states such as MAFLD and alcoholic fatty liver disease (AFLD). The semiquantitative grading system termed the NAFLD Activity Score (NAS) used by pathologists consists of grading histological sections for disease state based on the degree of steatosis, hepatocyte ballooning, and lobular inflammation. Figure 7, Figure 8 and Figure 9 quantify the average perimeters of lipid droplets from each diet group using WEKA Segmentation in FIJI (Supplementary Materials, Table S1) as a description of steatosis. The results for this study showed that LDs in female casein-fed groups (HFC and HFCN) increased in size from 6 to 12 months (Figure 7A,B and Figure 8A,B), at which time they plateaued until the termination of this study (Figure 9A,B). Overall, the average perimeter size was slightly larger in the HFC diet-fed group than the HFCN diet-fed group. In the beef-fed groups (HFB and HFBN), LDs increased in perimeter size from 6 to 12 months (Figure 7A,B and Figure 8A,B). At 18 months, the HFBN diet-fed females showed LDs that reduced slightly, while in the HFB diet-fed females, the LDs disappeared (Figure 9A,B). Overall, the males exhibited trends for LD size that were similar for all groups (Figure 7, Figure 8 and Figure 9), with the LDs gradually increasing until 18 months. There were two exceptions to this observation: the HFC diet-fed males demonstrated a smaller LD perimeter at 6 months (Figure 7A,C), and the HFBN diet-fed group had the greatest increase in LD size over the duration of this study (Figure 7A,C, Figure 8A,C and Figure 9A,C). While this study does not assess the severity of ballooning hepatocytes, it is worth noting that ballooning was present in all but three sections analyzed. Representative images of histological sections from each diet are located in Supplementary Figure S2.

### 3.10. Tumor Incidence and Type

**Figure 10:**
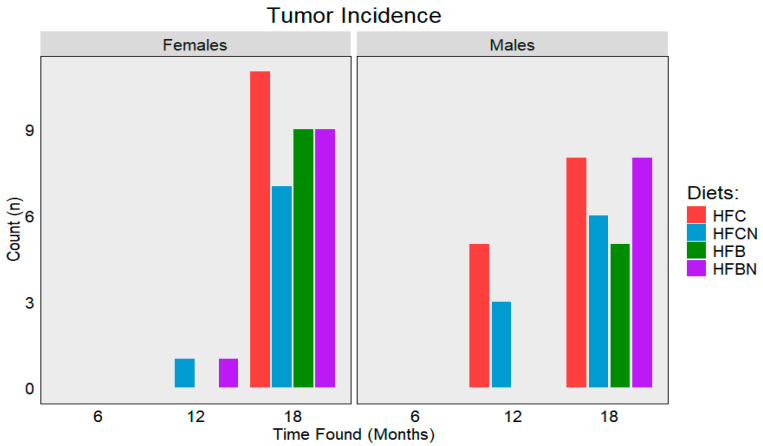
Total tumors observed during censorship points for each diet. Bar colors and diets: red = HFC, blue = HFCN, green = HFB, and purple = HFBN.

Over the course of this study, a total of 73 tumors from various origins were identified between all groups and sexes (Figure 10). Tumor incidence between the sexes was almost even, with females presenting with 38 (52%) tumors and the males presenting with 35 (48%) of the tumors. Where the results diverge is in the distribution of tumor type. In females, the HFC diet-fed group had the highest tumor incidence (11), followed by HFBN (10), HFCN (9), and HFB (8). Primary tumor types for females are listed in Table 3. Ovarian cancer was the most frequently identified within all dietary groups, except for the HFB diet-fed group, which displayed a higher incidence of mammary tumors. The third highest incidence in females was lung tumors, and liver tumor incidence was the lowest. In males, the HFC diet-fed group had the highest incidence (13), followed by HFCN (9), HFBN (8), and HFB (5). The results for males are listed in Table 4. Unlike the distribution of different cancers in the females, the males were almost exclusively liver.

For both sexes, the majority of tumors were recorded at ~18 months, and none were recorded prior to ~12 months. Tumor incidence was recorded from both the censored groups and non-censored individuals (died prior to collection), with non-censored individuals included in the total of the closest censored time point. In association with the incidences of liver tumors and increased fat mass, it is likely that there is some connection to liver lipid infiltration consistent with MAFLD.

## 4. Discussion

Previous studies have established the WD as one of the major contributors to the obesity epidemic [1,2]. Preclinical studies of DIO (animal models) tend to focus on acutely induced obesity rather than the chronic condition it is [5,6,7,8]. Furthermore, the central concern of these studies is an HF diet and the quantity of highly processed sugars, often ignoring protein source as uninvolved. The results from this study establish the importance of DPSs for both sexes, in a long-term mouse model of DIO. The major strengths of this study are the duration; allowing chronic obesity to develop; the use of both male and female mice in a strain where both sexes are susceptible to developing obesity (C3H/HeJ) [33]; and exploring “food as medicine” by modifying dietary protein components with AHE [7,27]. Overall, this study established that both DPSs and AHE are influential factors contributing to changes in longevity for C3H mice, and these influences change depending on sex. In females, this difference appears to be most attributed to DPSs as changes in mass composition are strongly impacted by HFD with beef or casein. Further, the addition of AHE emphasizes differences in mass composition as a result of DPSs in females. Female liver steatosis follows a similar trend demonstrating some reduction in LD size as the result of DPSs and DPSs with AHE. For males, dietary factors contributing to longevity appear more nuanced with AHE drastically improving longevity with a beef DPS but providing only a slight change to casein DPSs. In terms of mass composition, few significant changes were observed as the result of changed dietary components. Further, it appears that coupled with aging, DPSs are the major contributors to changes in liver LD size associated with steatosis.

AHE presents a novel method to address chronic low-grade metabolic acidosis, a condition strongly linked to the development of obesity and other metabolic diseases, like MAFLD [11,40]. Typical treatments of both chronic low-grade metabolic acidosis and obesity center around altering established dietary patterns [41,42]. The limiting factor to success using dietary interventions is almost always adherence. In fact, it has been stated that “dietary adherence is a more important factor in weight loss success than ‘type’ of diet” [41]. However, this is often the most difficult aspect of treatment for patients to follow. For example, adherence to the nationally recommended diet, The Dietary Approaches to Stop Hypertension (DASH), received an adherence score of 2.6 out of 9 in patients prescribed the diet by a physician [43]. In contrast, weekly interventions requiring little commitment, such as the use of GLP-1 receptor agonists for weight loss, demonstrate ≥ 85% adherence [44]. In terms of preclinical HFDs, the impacts of protein sources are regularly overlooked as demonstrated by the abundance of studies citing high-fat and high-sugar diets but not specifying protein, or if protein is discussed, it is in a particular quantity, not protein source [45,46,47]. In this study, it was demonstrated that the dietary protein can play a crucial, sometimes sex-based, role in the development of obesity. In addition, this study demonstrates modifications to dietary protein can alter health in yet unexplored ways. For example, recent work from our group showed that the gut microbiome was altered as a result of DPSs and AHE [27]. These changes included microbiome-based functions (e.g., glycine betaine transport, xenobiotic detoxification) that provide positive health benefits. Furthermore, AHE diets demonstrated decreases in gut microbiome diversity, which coincided with decreases in gut dysbiosis [27].

In this study, females fed AHE diets had improved longevity regardless of the protein source. However, we also observed DPS-dependent divergences in total mass over time, with increases in HFCN diet-fed females and decreases in HFBN diet-fed females. It is possible that increases in longevity due to AHE reflect the changes in the microbiome, and they are also supported by improvements to metabolic outcomes demonstrated by male C57bl/6 mice in a similar study [7]. Although the study by Menikdewella et al. did not observe improvements in females, this may be, in part, due to obesity resistance in female C57bl/6 mice fed an HF diet [7]. Major divergence is observed when assessing mass composition associated with aging. The AHE casein diet demonstrated greater decreases in fat and lean mass, while the AHE beef diet-fed mice showed only slight decreases in fat mass and an increase in lean mass. These findings have implications for muscle function in older females, and the benefits of this dietary approach need further study. When considering liver morphology and the development of liver steatosis by the analysis of LD size, larger LDs were observed in the females on casein diets, ± AHE to protein, compared with their beef counterparts. Livers from females consuming a beef DPS also demonstrated a decrease in LD size associated with aging. This finding also has important implications for aging females and dietary recommendations, especially when considering that this occurred in the context of the consumption of a high-fat diet. More studies are needed to understand the molecular impact of DPSs together and separately from AHE in the dietary protein.

Similar trends in the gut microbiome were reported in males and females with AHE [27]. However, sex-based differences were also observed in this study. In males, major improvements to longevity were observed as a result of AHE in beef protein, while almost no difference was seen between casein DPS ± AHE [27]. In our study, HFBN diet-fed males were the only group in the entire study (including females) to go from the lowest total mass at 6 months to the greatest total mass while aging (18 months). Importantly, all other male groups demonstrated an earlier onset of age-related decreases in total mass. Although few differences in mass composition were observed in males, the marked increase in lean mass prior to the onset of aging between HFBN diet-fed males and HBF diet-fed males suggests that this played a major role in survivability. These findings differ from those reported for C57bl/6, where AHE regardless of DPSs improved metabolic outcomes in males [7]. However, this previous study not only differed in the mouse strain but also the duration of the study (16 weeks), performing a well-established approach for DIO studies. While long-term studies such as ours are not always feasible, it is possible that the well-established C57bl/6 could be comparatively considered an acute model for DIO, and a long-term model for DIO would demonstrate divergences in longevity and total mass between HF diets ± AHE [7,33]. With regards to aging, the results showed that liver steatosis remained relatively stable for males consuming casein ± AHE. However, beef ± AHE demonstrated increases in LD size associated with age. LD size in male livers was larger compared to their female counterparts. This reflects current clinical findings denoting a sex dependent predisposition of males to developing MAFLD [48]. The molecular basis for this phenomenon may be related to muscle tissue function as differences in DPSs are supported in other contexts, like muscle building and even bone mass, and thus requires further attention [49,50].

Very little information is available exploring sexual dimorphism and DPSs. To date, no other studies were identified that modified this crucial dietary component to improve metabolic outcomes [51]. In terms of dietary protein and amino acid content, attempts have been made to associate particular AAs with chronic health disorders, like MAFLD or cerebrovascular disease [47,52]. While proteins containing high levels of methionine, homocysteine, and cysteine (red meats) are suspected of increasing the risk of the diseases mentioned above, there is yet to be conclusive evidence identifying these as causative [52]. In fact, based on our reported beneficial results with AHE beef incorporated into a high-fat diet (this study and [27]), this underscores the critical need to better understand protein's impacts on metabolic health.

HFDs are associated with the development of liver cancers and other tumors [4,53,54,55]. As cancer is considered a disease of aging and there is more evidence linking high-fat diet and obesity with earlier cancer incidence, it was of interest to analyze cancer development over diet, sex, and age using C3H/HeJ mice [55]. Tumor incidence displays an interesting trend with similar tumor numbers between each sex maintained on the same diet, except in HFB diet-fed females which demonstrated a higher incidence. This discrepancy is likely due to the strong impact of aging on cancer development as the HFB diet-fed males demonstrated much lower survivability than their female counterparts [56]. If expressed as incidence rate (tumors: individuals alive), the two HFB diet-fed sexes would more closely resemble each other. The incidence type also closely resembled that of humans, with males having a higher incidence of liver cancer than females [48]. Many studies point to the development of MAFLD and metabolic-associated steatohepatitis, two diseases shown to be more prevalent in men [48,57], as driving factors behind liver disease. However, one possibility is that liver cancer did not develop as readily in females, since estrogen is thought to play a protective role against liver tumor development [48]. In our study, large and well-vascularized liver tumors were observed in aged males, while less severe liver tumors were observed in aging females. These observations reflect human trends in aging for post-menopausal women who show a later onset of liver cancer, with less well-developed tumors than their male counterparts [33,48]. Moreover, the variety of cancers identified in females, some easily attributable to sex (e.g., mammary and ovarian), show that even with the protective effects of estrogen, aging is still a strong driver for cancer development [56].

The use of dietary component-based interventions, like AHE dietary protein, could open up a new avenue to increase adherence to a dietary intervention in a simple, yet effective way. It is demonstrated in this study and in Menikdewella et al. [7] that AHE (pH enhancement) in dietary proteins can provide nuanced improvements to metabolic dysfunction associated with obesity. The strengths of this study include the sample size used, as well as the length of time animals were assessed. Other strengths are the inclusion of both sexes, semi-automated tissue assessment, and the demonstration of a novel method of dietary intervention, which would not disrupt established dietary patterns. The limitations of this study include the lack of serum and blood glucose measurements. One other limitation, which will be addressed by future studies, is the lack of a full NAFLD Activity Score, a semiquantitative grading system used for assessing the progression of MAFLD [58]. Ongoing studies by our group are investigating tissue-specific and sex-dependent, metabolism-related changes with AHE protein in high-fat diets and cancer incidence. Future studies will elucidate specific mechanisms in AHE protein HF diets that give rise to this beneficial attenuation of DIO-related conditions.

## 5. Conclusions

Overall, this study demonstrates that dietary protein source ± ammonium hydroxide enhancement in an HFD is a major contributor to longevity, body mass, and attenuated metabolic-associated disease progression in a sex-dependent manner. Longevity was improved or unchanged with the addition of AHE in both males and females, demonstrating for the first time that AHE is effective as a potential approach to “Food as Medicine” to an established and continuing dietary pattern. It was also demonstrated that females may physiologically utilize a casein DPS to increase body mass (both fat and lean) and a beef DPS to decrease body mass (both fat and lean). Interestingly and importantly, our findings demonstrated that females physiologically benefited from beef DPS +AHE by decreasing fat mass and sustaining or slightly increasing lean mass with age. Males more effectively utilized beef DPS + AHE over other diets with respect to lean mass gain. These findings support others indicating that AHE can improve DIO, likely in a non-disruptive manner. Further exploration into key metabolic markers, such as glucose clearance and serum protein levels, is warranted to determine whether DIO models show benefits from AHE, especially in a long-term setting. With support from clinical studies, these findings may progress the field towards a novel approach to dietary interventions without the need to disrupt established dietary patterns.

## Figures and Tables

**Table 1:** Female mass composition compared with 4-week-old controls.

	Age (Mon)	(*n*)	Fat ± SE	% Fat	% ∆ Fat	Lean ± SE	% Lean	% ∆ Lean	Mass ± SE	% ∆ Total
Control	1	8	1.19 ± 0.24	6.90		15.31 ± 0.68	88.99		17.2 ± 0.59	
HFC	6	19	6.4 ± 0.56	21.11	437.82	15.92 ± 0.43	52.51	3.98	30.32 ± 1.11	76.28
	12	13	13.85 ± 2.02	35.47	1063.87	23.45 ± 0.56	60.05	53.17	39.05 ± 2.37	127.03
	18	8	7.83 ± 2.23	24.43	557.98	22.09 ± 0.68	68.92	44.28	32.05 ± 2.77	86.34
HFCN	6	17	7.76 ± 0.81	24.13	552.10	17.53 ± 0.52	54.51	14.50	32.16 ± 1.02	86.98
	12	14	16.43 ± 1.6	39.79	1280.67	23.82 ± 0.49	57.69	55.58	41.29 ± 2.02	140.06
	18	12	12.25 ± 2.09	33.23	929.41	23.58 ± 0.58	63.97	54.02	36.86 ± 2.7	114.30
HFB	6	18	9.08 ± 0.59	28.93	663.03	19.38 ± 0.49	61.74	26.58	31.39 ± 0.82	82.50
	12	10	9.37 ± 0.8	25.04	687.39	16.45 ± 0.67	43.96	7.45	37.42 ± 1.56	117.56
	18	9	9.59 ± 1.17	26.87	705.88	23.42 ± 0.53	65.62	52.97	35.69 ± 1.39	107.50
HFBN	6	22	7 ± 0.85	23.26	488.24	19.75 ± 0.46	65.64	29.00	30.09 ± 1.19	74.94
	12	14	10.88 ± 1.22	29.94	814.29	18.62 ± 0.57	51.24	21.62	36.34 ± 1.8	111.28
	18	10	5.77 ± 0.7	18.12	384.87	17.04 ± 0.98	53.50	11.30	31.85 ± 1.64	85.17

Note: Highlighted cells indicate maximum difference from controls for each mass type. Age is presented in months; Control (T0) indicates mice used after acclimatization period. “*n*” indicates the sample size for each row. Mass type ± SE indicates the average mass (g) and the standard error for each group at the specified time point. % mass type indicates the percentage of total mass each component makes up. % ∆ mass type indicates the % change which occurred from the start of this study until the specified time point.

**Table 2:** Male mass composition compared with 4-week-old controls.

	Age (Mon)	(*n*)	Fat ± SE	% Fat	% ∆ Fat	Lean ± SE	% Lean	% ∆ Lean	Mass ± SE	% ∆ Total
Control	1	8	1.44 ± 0.58	6.60		19.09 ± 2.14	87.65		21.78 ± 2.61	
HFC	6 Months	24	11.05 ± 0.48	26.63	667.36	28.58 ± 0.47	68.88	49.71	41.49 ± 0.91	90.50
	12 Months	15	9.35 ± 0.91	24.22	549.31	29.52 ± 0.59	76.46	54.64	38.61 ± 1.79	77.27
	18 Months	11	7.52 ± 1.21	20.91	422.22	27.27 ± 0.62	75.83	42.85	35.96 ± 1.76	65.11
HFCN	6 Months	16	11.1 ± 0.8	25.73	670.83	28.86 ± 0.52	66.90	51.18	43.14 ± 0.78	98.07
	12 Months	11	12.29 ± 0.93	28.29	753.47	29.82 ± 0.73	68.65	56.21	43.44 ± 1.7	99.45
	18 Months	10	8.14 ± 1.19	21.44	465.28	28.56 ± 0.58	75.22	49.61	37.97 ± 1.7	74.33
HFB	6 Months	18	9.39 ± 0.55	22.62	552.08	23.91 ± 0.5	57.60	25.25	41.51 ± 0.98	90.59
	12 Months	10	8.72 ± 1.54	23.14	505.56	27.81 ± 1.13	73.79	45.68	37.69 ± 2.29	73.05
	18 Months	4	8.31 ± 1.45	21.60	477.08	28.15 ± 0.53	73.15	47.46	38.48 ± 1.81	76.68
HFBN	6 Months	23	7.57 ± 0.86	18.81	425.69	30.98 ± 0.7	76.97	62.28	40.25 ± 0.69	84.80
	12 Months	13	10.67 ± 0.87	23.83	640.97	29.62 ± 0.37	66.16	55.16	44.77 ± 1.28	105.56
	18 Months	12	9.39 ± 1.04	23.12	552.08	30.14 ± 0.46	74.22	57.88	40.61 ± 1.35	86.46

Note: Highlighted cells indicate the maximum difference from controls for each mass type. Age is presented in months; Control (T0) indicates mice used after the acclimatization period. “*n*” indicates the sample size for each row. Mass type ± SE indicates the average mass (g) and the standard error for each group at the specified time point. % mass type indicates the percentage of total mass each component makes up. % ∆ mass type indicates the % change which occurred from the start of this study until the specified time point.

**Table 3:** Tumor distribution for female mice across each diet.

Female Tissue of Tumor Origin	HFC	HFCN	HFB	HFBN
Liver	2	2	1	1
Lung	4	2	3	4
Mammary			4	2
Ovary	5	4	1	3
Total	11	8	9	10

**Table 4:** Tumor distribution for male mice across each diet.

Male Tissue of Tumor Origin	HFC	HFCN	HFB	HFBN
Liver	13	9	4	8
Lung			1	
Total	13	9	5	8

## Data Availability

The full code and raw data are available at https://github.com/BenjaminBarr/HF-and-AHE-Diet/releases/tag/v1.1 (accessed on 8 August 2024). Other data are available upon reasonable request to the corresponding author.

## Supplementary Materials

The following supporting information can be downloaded at https://www.mdpi.com/article/10.3390/nu16162787/s1, Table S1: Diet formulations; Table S2: Effects of dietary components and sex on total mass and mass composition in C3H/HeJ mice; Figure S1: WEKA workflow; Figure S2: Liver lipid accumulation and distribution for each diet over time.
