# Supplementary material for: The Modification of Dietary Protein with Ammonium Hydroxide Enhancement Improves Longevity and Metabolic Outcomes in a Sex-Dependent Manner
Source: Nutrients. 2024 Aug 21;16(16):2787. doi: 10.3390/nu16162787 (PMC11357104; doi:10.3390/nu16162787)
Supplement: Supplementary file 1 [file nutrients-16-02787-s001.zip › nutrients-3151069-supplementary.pdf]

Supplementary Materials:

Table S1. Diet Formulations

	D20013103	D20013104	D20013105	D20013106
Product #	*46 kcal% Fat*<br>*High-Fat Diet*	*46 kcal% Fat*<br>*High-Fat Diet*	*46 kcal% Fat*<br>*High-Fat Diet*	*46 kcal% Fat*<br>*High-Fat Diet*
	AHE Beef (HFBN)	*non-AHE Beef (HFB)*	*AHE Casein (HFCN)*	*non-AHE Casein (HFC)*
Ingredient (g)				
Casein	0	0	0	200
Casein, "pH-Enhanced"	0	0	200	0
Beef, Cooked, Freeze Dried, AHE	352.42	0	0	0
Beef, Cooked, Freeze Dried, non-AHE	0	293.64	0	0
L-Cystine	3	3	3	3
Corn Starch	68.68	68.46	72.8	72.8
Maltodextrin 10	100	100	100	100
Sucrose	175.21	175.21	175.21	175.21
Cellulose	45.07	48.53	50	50
Soybean Oil	25	25	25	25
Lard	0	0	0	0
Beef Fat, Bunge	23.28	78.76	177.5	177.5

Mineral Mix S10026 (No Ca, P, K)	0	0	0	0
Mineral Mix S10026A (No Ca, P, K, Na, Cl)	5	5	5	5
Dicalcium Phosphate	13	13	13	13
Calcium Carbonate	5.5	5.5	5.5	5.5
Potassium Citrate, 1 H2O	16.5	16.5	16.5	16.5
Sodium Chloride	1.088	0.87	2.546	2.546
Vitamin Mix V10001	10	10	10	10
Choline Bitartrate	2	2	2	2
Cholesterol	0	0.02	0.49	0.49
Yellow Dye #5, FD&C	0	0.025	0.05	0
Red Dye #40, FD&C	0.025	0.025	0	0
Blue Dye #1, FD&C	0.025	0	0	0.05
Total	845.798	845.54	858.596	858.596
gm				
Protein	179.0	179.0	179.0	179.0
Carbohydrate	358.0	358.0	358.0	358.0
Fat	204.9	204.9	204.9	204.9

Fiber	50.0	50.0	50.0	50.0
Cholesterol	0.66	0.66	0.66	0.66
gm%				
Protein	21.2	21.2	20.8	20.8
Carbohydrate	42.3	42.3	41.7	41.7
Fat	24.2	24.2	23.9	23.9
Fiber	5.9	5.9	5.8	5.8
Cholesterol	0.08	0.08	0.08	0.08
kcals				
Protein	716.0	716.0	716.0	716.0
Carbohydrate	1432.1	1432.1	1432	1432
Fat	1844	1844.0	1844.1	1844.1
Total	3992.1	3992.1	3992.1	3992.1
kcal%				
Protein	18	18	18	18
Carbohydrate	36	36	36	36
Fat	46	46	46	46
Total	100	100	100	100
kcal/gm	4.7	4.7	4.6	4.6
Linoleic Acid, gm/kg Diet *(NRC Requirement is 6.8)*	20.8	21.3	21.9	21.9
Arginine, gm/kg Diet *(NRC Requirement is 3.0)*	13	13.6	6.9	6.9

Histidine, gm/kg Diet *(NRC Requirement is 2.0)*	6.2	6.8	5.2	5.2
Isoleucine, gm/kg Diet *(NRC Requirement is 4.0)*	8.7	9.2	8.7	8.7
Leucine, gm/kg Diet *(NRC Requirement is 7.0)*	15	15.8	18.3	18.3
Valine, gm/kg Diet *(NRC Requirement is 5.0)*	9.4	9.8	10.7	10.7
Threonine, gm/kg Diet *(NRC Requirement is 4.0)*	8.2	8.6	8.3	8.3
Lysine, gm/kg Diet *(NRC Requirement is 4.0)*	16.2	17.2	15.1	15.1
Methionine, gm/kg Diet *(NRC Requirement is 5.0; half can be Cystine)*	4.8	5.5	5.8	5.8
Cystine, gm/kg Diet *(NRC Requirement is - see above)*	5.5	5.9	3.6	3.6

Phenylalanine, gm/kg Diet *(NRC Requirement is 7.6; half can be Tyrosine)*	7.6	8	9.7	9.7
Tyrosine, gm/kg Diet *(NRC Requirement is - see above)*	6.2	6.5	10.5	10.5
Tryptophan, gm/kg Diet *(NRC Requirement is 1.0)*	2.2	2.4	2.4	2.4
Sodium (mg/kg diet)	1221	1222	1203	1203
Sodium (mg/3982.5 kcal)	1033	1033	1033	1033

Table S2. Effects of dietary components and sex on total mass and mass composition in C3H/HeJ mice.

Mass Type	Age (Months)	Statistic	Sex (S)	Enhancement (E)	Protein (P)	S x E	S x P	E x P	P x E x S
			Main Effects			Interactions			
Total Mass	6	*p*	7.89E-33	0.954394	0.163703	0.922302	0.540064	0.029344	0.934039
		F (1,149)	239.6619	0.003282	1.958963	0.009545	0.37716	4.840161	0.006873
	12	*p*	0.0616	0.021306	0.247243	0.041579	0.179465	0.827547	0.307383
		F (1,92)	3.580682	5.48775	1.355998	4.270961	1.82981	0.047729	1.053545
	18	*p*	0.00725	0.259076	0.620817	0.504826	0.198478	0.119761	0.152482
		F (1,68)	7.664648	1.295255	0.246974	0.449536	1.686289	2.482496	2.093903
Lean Mass	6	*p*	4.54E-59	1.96E-09	0.063961	0.002559	8.37E-08	0.000211	3.74E-07
		F (1,149)	723.8485	40.89183	3.483167	9.413513	31.78717	14.43633	28.29852
	12	*p*	3.04E-33	0.063879	7.01E-12	0.707949	2.32E-07	0.069888	0.876881
		F (1,92)	352.8885	3.517948	61.78907	0.141207	31.23316	3.363527	0.024136
	18	*p*	1.68E-23	0.656592	0.171235	1.56E-05	1.78E-05	0.0001	4.86E-05
		F (1,68)	229.9204	0.19944	1.912275	21.65467	21.31905	17.08209	18.827
Fat Mass	6	*p*	2.39E-05	0.088508	0.086444	0.385034	0.000735	0.010031	0.441302
		F (1,149)	19.02851	2.939666	2.978617	0.759028	11.88694	6.802228	0.596077
	12	*p*	0.006308	0.034009	0.001325	0.670566	0.039831	0.589414	0.982924
		F (1,92)	7.814894	4.631458	10.969	0.182107	4.347623	0.293314	0.000461
	18	*p*	0.515286	0.454434	0.372873	0.676306	0.073411	0.053904	0.056832
		F (1,68)	0.42778	0.566039	0.804645	0.175827	3.306498	3.847907	3.754103

Note: *p* and F values of three-way ANOVA to evaluate the main effect and their interaction at each time point. S, Sex (Female or Male); E, Enhancement (±AHE); P, Protein (casein or beef); x indicates interaction, highlighted values indicate significance.

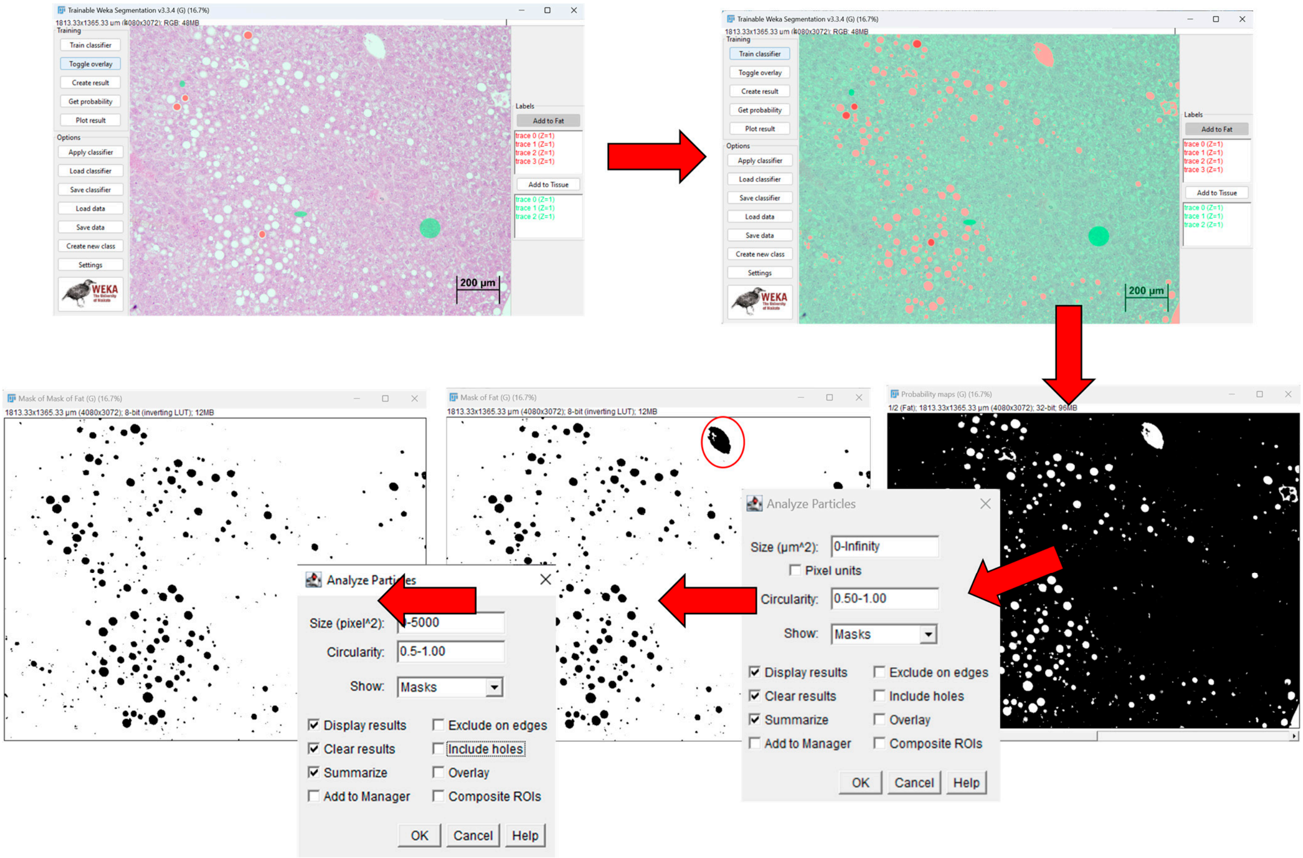

Figure S1. Representative images of windows during the WEKA workflow. A) Load Image into FIJI -> *Plugins* -> *Segmentation* -> *Trainable Weka Segmentation* -> *Settings* -> Change *Class Names* -> B) Select Examples of Lipid Droplets and Liver Tissue -> *Train Classifier* -> C)* Assess selections made by classifier using *Toggle Overlay* -> D) Improve classifier by adding or removing examples from Each class -> *Get Probability* -> *Image* -> *Type* -> *8-bit* -> *Image* -> *Stacks* -> *Stack to Images* -> *Adjust Lower Threshold* E) (generally ~200 for LD) -> *Analyze* -> *Analyze Particles* -> Set *Circularity* (0.5-1 for LD) -> *Ok.*

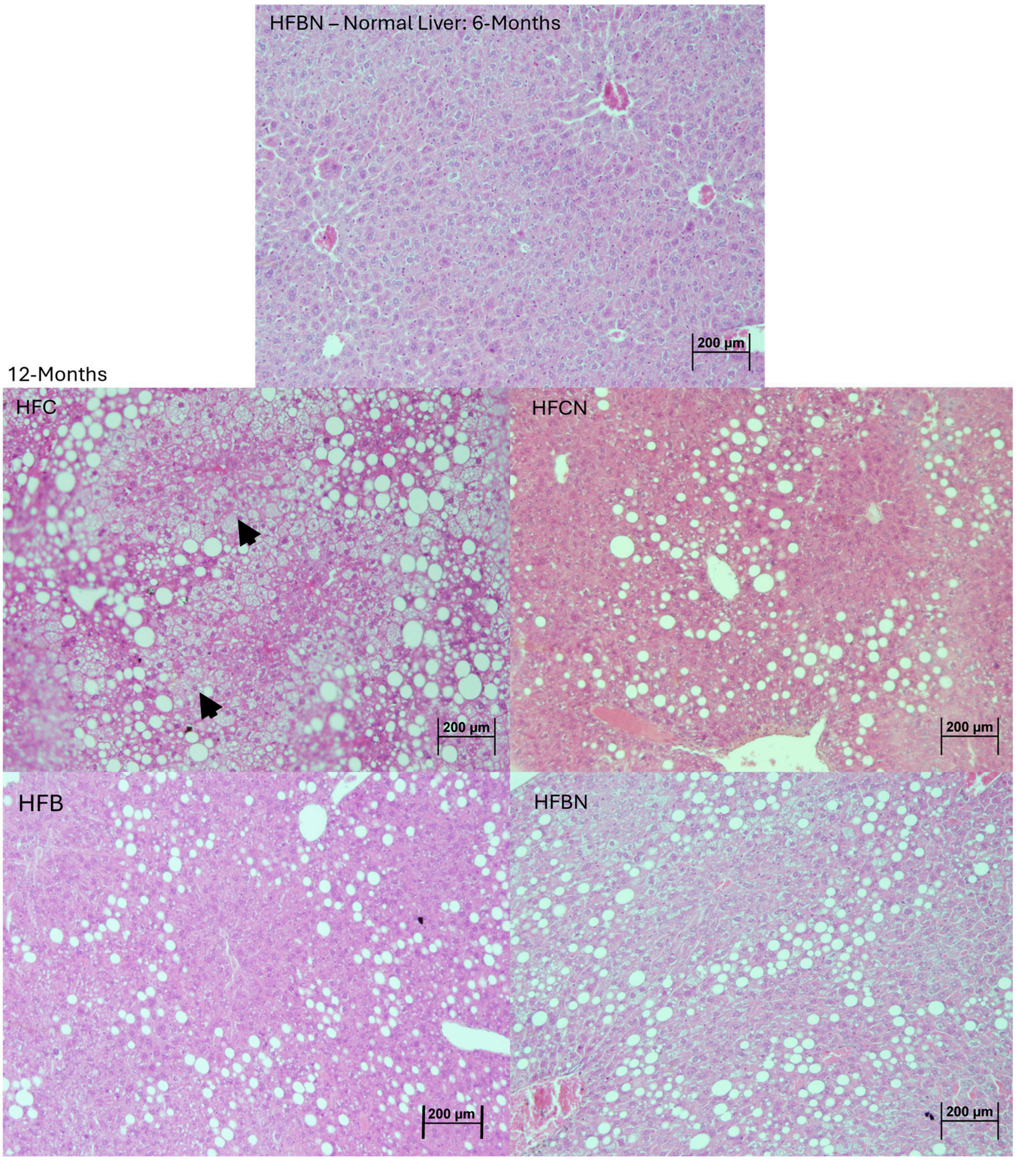

Figure S2. Liver lipid accumulation and distribution for each diet over time. Topmost centered image is an example of healthy liver from a HFBN male at 6-months. Representative liver sections from male livers at 12-months are below with varying degrees of steatosis. Profuse white circles are examples of lipid droplets assessed by the workflow. Black arrows indicate ballooning hepatocytes. White space with eosinophils illustrate blood vessels.
